# The impact of sarcopenia on the outcome of patients with left-sided colon and rectal cancer after curative surgery

**DOI:** 10.1186/s12885-023-11073-0

**Published:** 2023-07-10

**Authors:** Qi Li, Tailai An, Jianbin Wu, Weiqi Lu, Yan Wang, Jia Li, Lina Yang, Yiqi Chen, Lizhu Lin, Zhenjiang Yang

**Affiliations:** 1 Department of Medical Oncology, The Fourth Clinical Medical College of Guangzhou University of Chinese Medicine, Fuhua Road 1, Futian District, Shenzhen, 518033 Guangdong People’s Republic of China; 2 Department of Hepatobiliary and Pancreatic Surgery, Shenzhen People’s Hospital, Dongmen North Road 1017, Luohu District, Shenzhen, 518000 Guangdong People’s Republic of China; 3 Department of Gastrointestinal Surgery, The First Hospital, Guangzhou University of Traditional Chinese Medicine, Jichang Road 16, Baiyun District, Guangzhou, 510400 Guangdong People’s Republic of China; 4 Department of Radiology, Shenzhen People’s Hospital, Dongmen North Road 1017, Luohu District, Shenzhen, 518000 Guangdong People’s Republic of China; 5 The First Department of Surgery, The Fourth Clinical Medical College of Guangzhou University of Chinese Medicine, Fuhua Road 1, Futian District, Shenzhen, 518033 Guangdong People’s Republic of China; 6 Department of Medical Oncology, The First Affiliated Hospital, Guangzhou University of Traditional Chinese Medicine, Jichang Road 16, Baiyun District, Guangzhou, 510400 Guangdong People’s Republic of China

**Keywords:** Sarcopenia, Left-sided colon cancer and rectal cancer, Psoas muscle index, Short-term and long-term outcome

## Abstract

**Background:**

The impact of sarcopenia on the outcome of patients with left-sided colon and rectal cancer has not been exhaustively investigated. Thus, the present study was performed to evaluate the effect of sarcopenia on the outcome of patients with left-sided colon and rectal cancer.

**Methods:**

Patients with pathologically diagnosed stage I, II and III left-sided colon or rectal cancer who had undergone curative surgery between January 2008 and December 2014 were retrospectively reviewed. The psoas muscle index (PMI) identified by 3D-image analysis of computed tomographic images was the criterion used to diagnose sarcopenia. The cut-off value recommended by Hamaguchi (PMI value < 6.36 cm^2^/m^2^ for men and < 3.92 cm^2^/m^2^ for women) was adopted to confirm the diagnosis of sarcopenia. According to the PMI, each patient was divided into the sarcopenia group (SG) or the nonsarcopenia group (NSG). Then, the SG was compared with the NSG in terms of postoperative outcomes.

**Results:**

Among the 939 patients included, 574 (61.1%) were confirmed to have preoperative sarcopenia. Initially, it was demonstrated that the SG was not significantly different from the NSG in terms of most baseline characteristics except for a lower body mass index (BMI) (*P* < 0.001), a larger tumour size (*P* < 0.001) and more weight loss (more than 3 kg in the last three months) (*P* = 0.033). The SG had a longer hospital stay after surgery (*P* = 0.040), more intraoperative blood transfusions (*P* = 0.035), and higher incidence of anastomotic fistula (*P* = 0.027), surgical site infection (SSI) (*P* = 0.037) and hypoalbuminemia (*P* = 0.022), 30-day mortality (*P* = 0.042) and 90-day mortality (*P* = 0.041). The SG had significantly worse overall survival (OS) (*P* = 0.016) and recurrence-free survival (RFS) (*P* = 0.036) than the NSG. Subsequently, Cox regression analysis revealed that preoperative sarcopenia was an independent predictive factor for worse OS (*P* = 0.0211, HR = 1.367, 95% CI: 1.049–1.782) and RFS (*P* = 0.045, HR = 1.299, 95% CI: 1.006–1.677).

**Conclusion:**

Preoperative sarcopenia adversely affects the outcome of patients with left-sided colon and rectal cancer, and preoperative nutrition supplementation may help us improve their long-term and short-term outcomes.

## Introduction

Colorectal cancer (CRC) is the third most common cancer worldwide and causes the second most cancer-related deaths [1]. Left-sided colon cancer and rectal cancer are the most commonly diagnosed. Over the past few decades, the survival of patients with CRC has been remarkably improved due to the popularization of the multidisciplinary teamwork (MDT) mode and the application of more treatment modalities.

Many studies have reported the significance of nutritional status among patients with CRC. Ashna Gupta reported that preoperative malnutrition in patients with colorectal cancer is associated with several postoperative consequences and poorer prognosis [2]. Moreover, malnutrition was more commonly encountered among patients with CRC. They argued that reasonable nutritional supplementation was of paramount significance to achieve favourable short-term outcomes and long-term survival [2]. As a unique type of malnutrition, sarcopenia has been evaluated among various cancers. According to a review by Papadopoulou SK, a progressive loss of skeletal muscle mass and loss of muscle function is defined as sarcopenia, and sarcopenia is currently considered as a prevalent health problem among elderly patients [3]. Two types of sarcopenia, primary and secondary sarcopenia have been proposed. Age-associated loss of muscle mass is defined as primary sarcopenia, while loss of muscle mass secondary to pathogenic diseases is defined as secondary sarcopenia [4, 5]. In an ageing society, sarcopenia among patients diagnosed with cancer not only includes secondary sarcopenia but also age-associated primary sarcopenia, but sometimes both types exist. In most studies, primary sarcopenia is not distinguished from secondary sarcopenia, and as a matter of fact, it is not easy to differentiate primary sarcopenia from secondary sarcopenia. Sarcopenia has been reported to lead to worse prognosis [6], especially for those with malignant tumours [7–11]. Similarly, sarcopenia has also been evaluated in CRC in a few studies, and sarcopenia has been identified as a negative prognostic factor for CRC [12, 13].

However, studies investigating the prognostic significance of preoperative sarcopenia among CRC patients are still scarce. Previous studies have included fewer patients. Additionally, it has been reported that right-sided colon cancer is significantly different from left-sided colon and rectal cancer in terms of long-term prognosis and cancer biology [14–16]. However, to the best of our knowledge, studies investigating the prognostic significance of sarcopenia among patients with left-sided colon or rectal cancer remain scarce. Therefore, the present study was performed to investigate the impacts of preoperative sarcopenia on the short-term and long-term outcomes of patients with left-sided colon or rectal cancer.

## Methods

### Patients and ethical approval

Patients with pathologically diagnosed left-sided colon or rectal cancer who had undergone curative surgery between January 2008 and December 2014 at The Department of Gastrointestinal Surgery, The First Affiliated Hospital, Guangzhou University of Chinese Medicine were retrospectively reviewed. However, patients suffering from complete obstruction, perforation and hemorrhage had been excluded from this study since these patients were usually dealt with by emergent surgeries. And emergent surgeries were more likely to result in higher morbidity rate. Before surgery, all the patients, especially those diagnosed with rectal cancer, were discussed by experts of different disciplines (i.e., MDT mode). Patients with rectal cancer routinely received magnetic resonance imaging (MRI) to assess the depth of invasion and lymph node metastasis. Preoperatively, for patients with locally advanced rectal cancer identified by MRI, medical oncologists and radiotherapists were consulted to assess indications and contraindications for neoadjuvant chemotherapy and radiotherapy. Similarly, after curative surgery, medical oncologists and radiotherapists were consulted to evaluate the adjuvant chemotherapy and radiotherapy given. The exclusion criteria adopted in this study were as follows: palliative surgery, systematic inflammation due to aetiologies other than colorectal cancer, insufficient follow-up information, synchronous or heterochronous colorectal cancer and malignant tumours of other organs. This study was approved by The Ethics Committee, The First Affiliated Hospital, Guangzhou University of Chinese Medicine (approval number: K2022-077). All the patients included in this study provided informed consent in written form. The following variables were included in this study: body mass index (BMI), age, gender, hypertension, American Society of Anesthesiologists (ASA) score, comorbidity, Charlson Comorbidity Index (CCI) [17, 18], weight loss (more than 3 kg in the last three months), intestinal obstruction (preoperative), tumour size, tumour location, gross morphology, tumour grading, histological component, lymphovascular invasion, perineural invasion, depth of invasion, lymph node metastasis, the number of harvested lymph nodes, Union Internationale Against cancer and American joint Committee on cancer(AJCC/UICC) stage, CEA, CA19.9, adjuvant chemotherapy, and postoperative complications. Postoperative complications were graded by the Clavien Dindo system [19]. The present study was in compliance with the Declaration of Helsinki during the whole process [20].

### Measurement of the psoas muscle index (PMI) and definition of sarcopenia

**Fig. 1:**
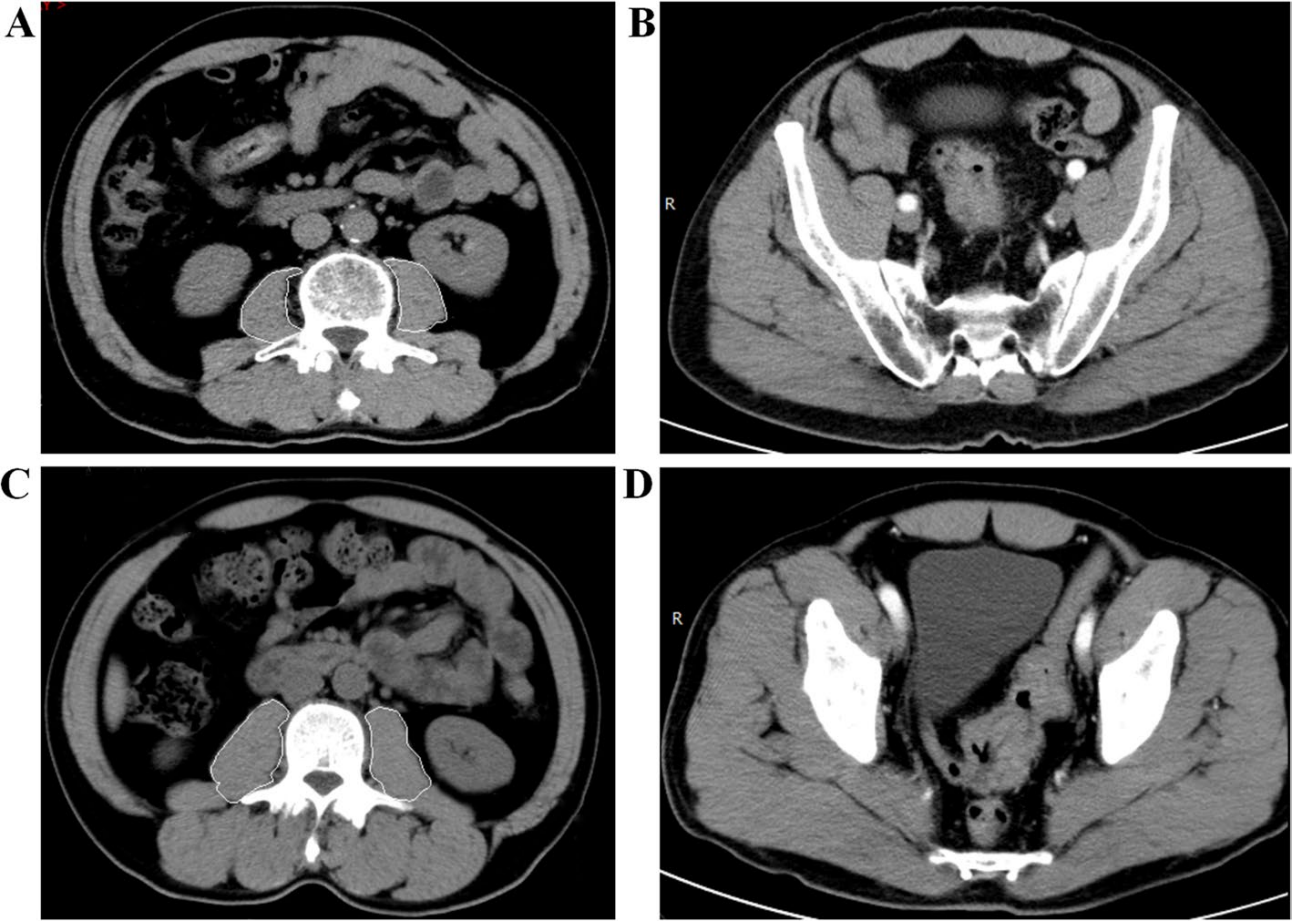
**A** Measuring the cross-sectional area of the psoas muscles at the level of the third lumbar vertebra for a patient with sarcopenia and sigmoid colon cancer. **B** Representative CT image of a patient diagnosed with sigmoid colon cancer and sarcopenia.** C** Measuring the cross-sectional area of the psoas muscles at the level of the third lumbar vertebra for a patient with sigmoid colon cancer but without sarcopenia. **D** Representative CT image of a patient diagnosed with sigmoid colon cancer but without sarcopenia

The cross-sectional areas of the psoas muscles at the level of the third lumbar vertebra (L3) on computed tomography (CT) were measured using IntelliSpace Portal (Version 10, Philips Health System, Best, Netherlands; Licence number 85954) (Fig. 1A-D). The PMI of each patient was obtained by dividing the area of the psoas muscle by the square of the height (m). Hamaguchi proposed that the PMI should be adopted as a diagnostic criterion for sarcopenia, and he proposed that a PMI < 6.36 cm^2^/m^2^ for male patients and a PMI < 3.92 cm^2^/m^2^ for female patients should be adopted to diagnose sarcopenia [21]. Similarly, the criteria proposed by Hamaguchi et al. were also adopted in this study. According to the PMI, each patient was assigned to the sarcopenia group (SG) or nonsarcopenia group (NSG).

### Statistical analysis

Continuous variables were compared using the t test or Wilcoxon signed-rank test, while categorical variables were compared by Fisher’s exact test. Overall survival (OS) was defined as the time length between curative surgery and death regardless of the cause, while the duration between curative surgery and cancer recurrence was recorded as recurrence-free survival (RFS). The Kaplan‒Meier method was used to calculate OS and RFS. The statistical significance of each comparison between SG and NSG was identified by the log-rank test. Cox regression analysis was performed to identify independent predictive factors for OS and RFS. The SG was compared with the NSG in terms of preoperative variables and postoperative outcomes. All the tests performed in this study were two-sided, and *P* values less than 0.05 were considered statistically significant. The aforementioned statistical analyses were accomplished by the Statistical Product and Service Solutions (SPSS) software package (Version 22, IBM, Armonk, NY, USA).

## Results

### Baseline characteristics

The baseline characteristics of the SG and NSG are summarized in Table 1. The SG included significantly more patients who had a remarkably lower body mass index, which was consistent with previous studies [22, 23]. The SG included 574 patients, while there were 365 patients in the NSG. The SG was not significantly different from the NSG in terms of most of the baseline characteristics except for a significantly lower BMI (*P* < 0.001), a larger tumour size (*P* < 0.001), and more weight loss (more than 3 kg in the last three months) (*P* = 0.033).

**Table 1:** Comparisons between the SG and NSG regarding baseline characteristics

Characteristics	No	Sarcopenia		χ^2^/t	*P*
		No(*N* = 365)	Yes(*N* = 574)	Value	Value
BMI		22.24 ± 2.98	21.52 ± 2.45	3.907	0.000^a^
Age		57.76 ± 12.89	58.65 ± 12.62	-1.048	0.295
60y	470(50.1)	187(51.2)	283(49.3)	0.332	0.564
≥ 60y	469(49.9)	178(48.8)	291(50.7)		
Gender				0.290	0.590
Male	553(58.9)	211(57.8)	342(59.6)		
Female	386(41.1)	154(42.2)	232(40.4)		
Hypertension				1.589	0.207
No	838(89.2)	321(87.9)	517(90.1)		
Yes	101(10.8)	44(12.1)	57(9.9)		
ASA				4.792	0.188
1	258(27.5)	112(30.7)	146(25.4)		
2	585(62.3)	223(61.1)	362(63.1)		
3	92(9.8)	29(7.9)	63(11.0)		
4	4(0.4)	1(0.3)	3(0.5)		
Charlson Comorbidity Index				0.986	0.805
No comorbidity (0)	386(41.1)	156(42.8)	230(40.1)		
Few comorbidities (1–2)	451(48.0)	173(47.4)	278(48.4)		
Moderate number of comorbidities (3–4)	85(9.1)	30(8.2)	55(9.6)		
High number of comorbidities (≥ 5)	17(1.8)	6(1.6)	11(1.9)		
Weight loss (3 kg in the last 3 months)				4.526	0.033^a^
No	515(54.8)	216(59.2)	299(52.1)		
Yes	424(45.2)	149(40.8)	275(47.9)		
Intestinal obstruction				0.002	0.963
No	870(92.7)	338(92.6)	532(92.7)		
Yes	69(7.3)	27(7.4)	42(7.3)		
Tumour size				18.851	0.000^a^
< 5 cm	480(51.1)	219(60.0)	261(45.5)		
≥ 5 cm	459(48.9)	146(40.0)	313(54.5)		
Location				1.851	0.604
Rectum	569(60.6)	230(63.0)	339(59.1)		
Sigmoid colon	289(30.8)	104(28.5)	185(32.2)		
Descending colon	60(6.4)	22(6.0)	38(6.6)		
Splenic flexure	21(2.2)	9(2.5)	12(2.1)		
Gross morphology				3.412	0.182
Massive	306(32.6)	109(29.9)	197(34.3)		
Ulcerative	530(56.4)	209(57.3)	321(55.9)		
Infiltrative	103(11.0)	47(12.9)	56(9.8)		
Tumour grading				0.970	0.325
G2/G3	874(93.1)	336(92.1)	538(93.7)		
G1	65(6.9)	29(7.9)	36(6.3)		
Component				1.730	0.421
Adenocarcinoma	899(95.7)	346(94.8)	553(96.3)		
Mucinous	32(3.4)	16(4.4)	16(2.8)		
Siglet-ring carcinoma	8(0.9)	3(0.8)	5(0.9)		
Lymphovascular invasion				2.308	0.129
No	890(94.8)	351(96.2)	539(93.9)		
Yes	49(5.2)	14(3.8)	35(6.1)		
Perineural invasion				1.715	0.190
No	922(98.2)	361(98.9)	561(97.7)		
Yes	17(1.8)	4(1.1)	13(2.3)		
T				7.720	0.052
T1	37(3.9)	19(5.2)	18(3.1)		
T2	160(17.0)	69(18.9)	91(15.9)		
T3	372(39.6)	151(41.4)	221(38.5)		
T4	370(39.4)	126(34.5)	244(42.5)		
N				0.129	0.937
N0	553(58.9)	214(58.6)	339(59.1)		
N1	244(26.0)	97(26.6)	147(25.6)		
N2	142(15.1)	54(14.8)	88(15.3)		
AJCC/UICC				3.063	0.216
I	162(17.3)	72(19.7)	90(15.7)		
II	392(41.7)	143(39.2)	249(43.4)		
III	385(41.0)	150(41.1)	235(40.9)		
CEA level(µg/L)				0.042	0.838
< 5	616(65.6)	238(65.2)	378(65.9)		
≥ 5	323(34.4)	127(34.8)	196(34.1)		
CA19.9				0.002	0.962
< 37	811(86.4)	315(86.3)	496(86.4)		
≥ 37	128(13.6)	50(13.7)	78(13.6)		
Chemotherapy				0.283	0.595
No	476(50.7)	189(51.8)	287(50.0)		
Yes	463(49.3)	176(48.2)	287(50.0)		
NC N/Y	234(50.5)/229(49.5)	83(47.2)/93(52.8)	151(52.6)/136(47.4)		

*NC N/Y* neoadjuvant chemotherapy No/Yes; ^a^*P*＜0.05

### Short-term outcomes

Subsequently, we compared the SG and NSG regarding intraoperative and postoperative outcomes, the results of which demonstrated that the SG had more intraoperative blood transfusions (*P* = 0.035), a higher incidence of hypoalbuminemia (*P* = 0.022), anastomotic fistula (*P* = 0.027) and surgical site infection (SSI) (*P* = 0.037) and a longer hospital stay (*P* = 0.040) (Table 2). Additionally, it was revealed that SG (19, 12–92) was not significantly different from NSG (19, 12–55) regarding the number of harvested lymph nodes (Z = -0.790, *P* = 0.430). Since anastomotic fistula was the most terrifying complications after curative surgeries for CRC, we then compared SG and NSG regarding re-operation rate after anastomotic fistula. Of the 38 patients suffering from anastomotic fistula in SG, 12 ones underwent reoperation while 4 patients of the 12 ones in NSG suffering from anastomotic fistula had undergone reoperation.

**Table 2:** Comparisons between the SG and NSG regarding intraoperative and postoperative outcomes

Characteristics	No	Sarcopenia		χ^2^/t	*P*
		No(*N* = 365)	Yes(*N* = 574)	Value	Value
Operation time		218.27 ± 85.16	220.36 ± 86.73	-0.363	0.717
Hospitalization after surgery		10.95 ± 7.18	12.00 ± 8.36	-2.061	0.040^a^
Intraoperative blood transfusion					
No	828(88.2)	332(91.0)	496(86.4)	4.427	0.035^a^
Yes	111(11.8)	33(9.0)	78(13.6)		
Colostomy				1.830	0.176
No	838(89.2)	332(91.0)	506(88.2)		
Yes	101(10.8)	33(9.0)	68(11.8)		
Abdominal infection				1.277	0.258
No	914(97.3)	358(98.1)	556(96.9)		
Yes	25(2.7)	7(1.9)	18(3.1)		
C-D I/II/IIIa/IIIb/IVa/IVb/V	0/24/1/0/0/0/0	0/7/0/0/0/0/0	0/17/1/0/0/0/0		
Pulmonary infection				0.197	0.657
No	924(98.4)	360(98.6)	564(98.3)		
Yes	15(1.6)	5(1.4)	10(1.7)		
C-D I/II/IIIa/IIIb/IVa/IVb/V	3/6/2/0/0/0/4	2/2/1/0/0/0/0	1/4/1/0/0/0/4		
Urinary infection				0.634	0.426
No	925(98.5)	361(98.9)	564(98.3)		
Yes	14(1.5)	4(1.1)	10(1.7)		
C-D I/II/IIIa/IIIb/IVa/IVb/V	13/1/0/0/0/0/0	4/0/0/0/0/0/0	9/1/0/0/0/0/0		
Urinary retention				3.060	0.080
No	926(98.6)	363(99.5)	563(98.1)		
Yes	13(1.4)	2(0.5)	11(1.9)		
C-D I/II/IIIa/IIIb/IVa/IVb/V	4/9/0/0/0/0/0	1/1/0/0/0/0/0	3/8/0/0/0/0/0		
Ureter injury				0.753	0.385
No	934(99.5)	364(99.7)	570(99.3)		
Yes	5(0.5)	1(0.3)	4(0.7)		
C-D I/II/IIIa/IIIb/IVa/IVb/V	0/0/2/3/0/0/0	0/0/0/1/0/0/0	0/0/2/2/0/0/0		
Surgical site infection				4.348	0.037^a^
No	920(98.0)	362(99.2)	558(97.2)		
Yes	19(2.0)	3(0.8)	16(2.8)		
C-D I/II/IIIa/IIIb/IVa/IVb/V	6/8/0/5/0/0/0	1/1/0/1/0/0/0	5/7/0/4/0/0/0		
Abdominal haemorrhage				3.579	0.059
No	934(99.5)	361(98.9)	573(99.8)		
Yes	5(0.5)	4(1.1)	1(0.2)		
C-D I/II/IIIa/IIIb/IVa/IVb/V	0/1/1/1/0/0/2	0/1/1/1/0/0/1	0/0/0/0/0/0/1		
Postoperative intestinal obstruction				0.240	0.624
No	921(98.1)	357(97.8)	564(98.3)		
Yes	18(1.9)	8(2.2)	10(1.7)		
C-D I/II/IIIa/IIIb/IVa/IVb/V	3/12/0/3/0/0/0	1/6/0/1/0/0/0	2/6/0/2/0/0/0		
DVT				0.047	0.828
No	932(99.3)	362(99.2)	570(99.3)		
Yes	7(0.7)	3(0.8)	4(0.7)		
C-D I/II/IIIa/IIIb/IVa/IVb/V	0/3/2/0/0/0/2	0/2/1/0/0/0/0	0/1/1/0/0/0/2		
Anastomotic fistula				4.915	0.027^a^
No	889(94.7)	353(96.7)	536(93.4)		
Yes	50(5.3)	12(3.3)	38(6.6)		
C-D I/II/IIIa/IIIb/IVa/IVb/V	0/9/18/16/0/0/7	0/3/4/4/0/0/1	0/6/14/12/0/0/6		
Hypoalbuminaemia				5.216	0.022^a^
No	814(86.7)	328(89.9)	486(84.7)		
Yes	125(13.3)	37(10.1)	88(15.3)		
30-day mortality				4.154	0.042^a^
No	928(98.8)	364(99.7)	564(98.3)		
Yes	11(1.2)	1(0.3)	10(1.7)		
90-day mortality				4.184	0.041^a^
No	924(98.4)	363(99.5)	561(97.7)		
Yes	15(1.6)	2(0.5)	13(2.3)		

*C–D* Clavien‒Dindo classification; ^a^*P*＜0.05

Since 30-day mortality and 90-day mortality is the most important parameter indicating safety and quality of curative surgery, we then assessed the impacts of sarcopenia on 30-day mortality and 90-day mortality and it was revealed that SG had significantly higher 30-day mortality (*P* = 0.042) and 90-day mortality (*P* = 0.041). For the 11 patients experiencing 30-day mortality, 4 ones died due to anastomotic fistula while anastomotic fistula caused 7 deaths occurring within 90 days after surgery.

### Impacts of sarcopenia on chemotherapy-related adverse effects

According to Chinese guidelines, patients with stage III CRC or stage II CRC but with high risk factors (such as LVI and PNI) should undergo chemotherapy. Then we assessed the impacts of sarcopenia on incidence of chemotherapy-related adverse effects and revealed that patients with sarcopenia were significantly more likely to suffer from anemia (*P* = 0.020), mucositis or stomatitis (*P* = 0.043) and alopecia (*P* = 0.024) (Table 3).

**Table 3:** Impacts of sarcopenia on chemotherapy-related adverse effects

Adverse effects	Non-sarcopenia(176)			Sarcopenia(287)			χ^2^	*P*
	0	1–2	3–4	0	1–2	3–4		
Leukopenia	99(56.2)	77(43.8)	0(0)	171(59.6)	115(40.1)	1(0.3)	1.177	0.555
Anemia	121(68.8)	55(31.3)	0(0)	167(58.2)	114(39.7)	6(2.1)	7.781	0.020
Thrombocytopenia	163(92.6)	13(7.4)	0(0)	263(91.6)	24(8.4)	0(0)	0.141	0.707
Nausea	77(43.8)	99(56.2)	0(0)	121(42.2)	166(57.8)	0(0)	0.113	0.737
Vomiting	163(92.6)	13(7.4)	0(0)	263(91.7)	23(8.0)	1(0.3)	0.680	0.712
Anorexia	82(46.6)	94(53.4)	0(0)	130(45.3)	54(53.7)	3(1.0)	1.881	0.390
Mucositis/Stomatitis	168(95.5)	18(4.5)	0(0)	258(89.9)	22(7.7)	7(2.4)	6.296	0.043
Taste alteration (Dysgeusia)	160(90.9)	16(9.1)	0(0)	264(92.0)	23(8.0)	0(0)	0.164	0.685
Neuropathy: cranial-smell	133(75.6)	43(24.4)	0(0)	213(74.2)	74(25.8)	0(0)	0.106	0.745
Diarrhea	152(86.4)	24(13.6)	0(0)	243(84.7)	42(14.6)	2(0.7)	1.339	0.512
Constipation	145(87.3)	21(12.7)	0(0)	236(82.2)	51(17.8)	0(0)	2.062	0.151
Fatigue (malaise)	133(75.6)	42(23.8)	1(0.6)	213(74.3)	73(25.4)	1(0.3)	0.257	0.879
Mood alteration: anxiety/Depression	175(99.4)	1(0.6)	0(0)	285(99.3)	2(0.7)	0(0)	0.028	0.867
Alopecia	161(91.5)	15(8.5)	0(0)	240(83.6)	41(14.3)	6(2.1)	7.452	0.024
Neuropathy: sensory	167(94.9)	9(5.1)	0(0)	274(95.5)	13(4.5)	0(0)	0.082	0.774
Insomnia	151(85.8)	24(13.6)	1(0.6)	242(84.4)	44(15.3)	1(0.3)	0.363	0.834

### Long-term outcomes: survival analysis

**Fig. 2:**
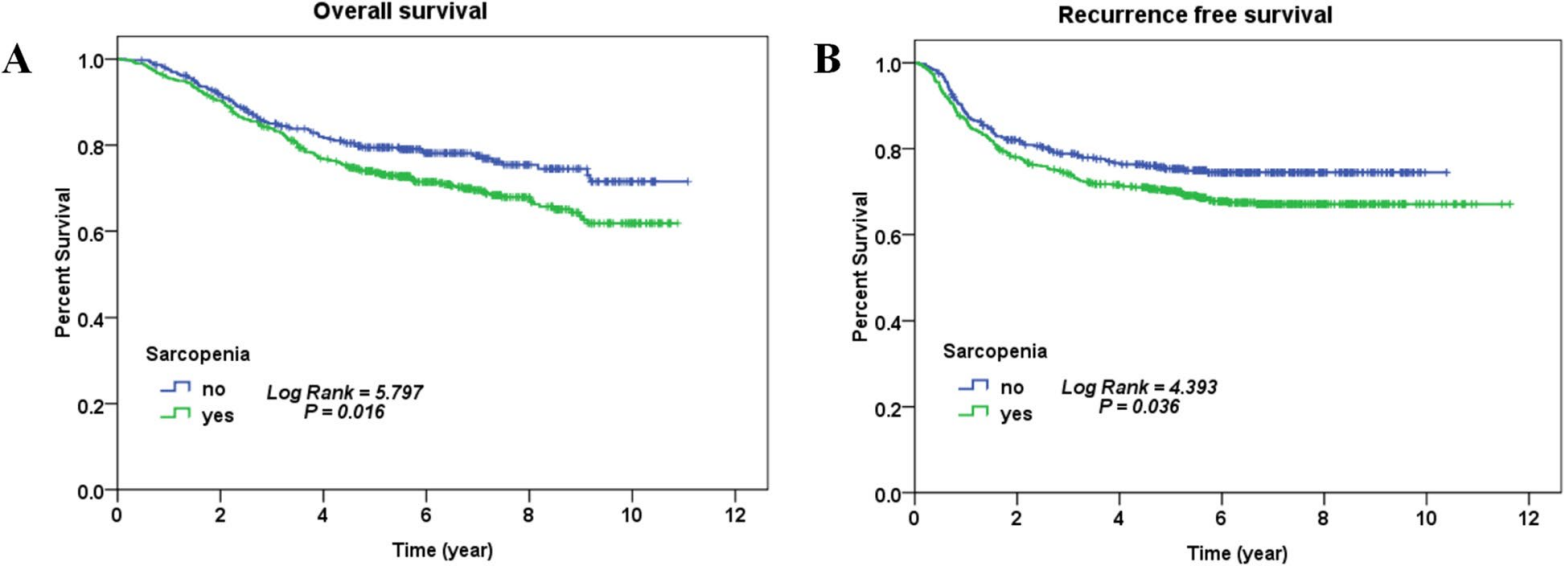
**A** Kaplan‒Meier analysis was performed to assess the impacts of sarcopenia on overall survival. **B** Kaplan‒Meier analysis was performed to assess the impacts of sarcopenia on recurrence-free survival

The Kaplan‒Meier method was used to calculate OS and RFS. The overall five-year OS rates and five-year RFS rates were 75.8% and 72.2%, respectively. The five-year OS rate of the SG was 73.6%, while that for the NSG was 79.4%. The five-year RFS rate of the SG was 70.2%, while that for the NSG was 75.3%. Survival curves were plotted by Kaplan‒Meier analysis, revealing that the SG was associated with significantly worse OS (*P* = 0.016) (Fig. 2A) and RFS (*P* = 0.036) (Fig. 2B).

To further evaluate the impacts of sarcopenia on OS and RFS, we then performed Cox regression analysis to demonstrate whether sarcopenia independently affected OS and RFS. Initially, univariate Cox regression analysis was performed to identify factors significantly associated with OS, demonstrating that age (*P* = 0.003, HR = 1.448, 95% CI: 1.131–1.854), tumour location (*P* = 0.003, HR = 0.745, 95% CI: 0.611–0.907), tumour grading (*P* = 0.006, HR = 0.345, 95% CI: 0.163–0.732), histological component (*P* < 0.001, HR = 2.504, 95% CI: 1.778–3.526), depth of invasion (*P* < 0.001, HR = 1.681, 95% CI: 1.412–2.001), lymph node metastasis (*P* < 0.001, HR = 2.087, 95% CI: 1.799–2.422), AJCC/UICC stage (*P* < 0.001, HR = 2.269, 95% CI: 1.856–2.774), lymphovascular invasion (*P* < 0.001, HR = 3.137, 95% CI: 2.117–4.648), perineural invasion (*P* < 0.001, HR = 4.311, 95% CI: 2.465–7.540), adjuvant chemotherapy (*P* = 0.006, HR = 0.708, 95% CI: 0.553–0.906), CEA (*P* < 0.001, HR = 1.598, 95% CI: 1.249–2.044), CA19.9 (*P* < 0.001, HR = 2.024, 95% CI: 1.505–2.722) and sarcopenia (*P* = 0.017, HR = 1.379, 95% CI: 1.060–1.794) were significantly associated with OS (Table 4). Then, these variables that were significantly associated with OS, as proven by the univariate Cox regression analysis, were included in the multivariate Cox regression analysis to identify independent predictive factors for OS, and the multivariate Cox regression analysis demonstrated that age (*P* < 0.001, HR = 1.622, 95% CI: 1.258–2.091), tumour location (*P* = 0.001, HR = 0.714, 95% CI: 0.585–0.871), histological component (*P* = 0.001, HR = 1.799, 95% CI: 1.263–2.561), depth of invasion (*P* = 0.012, HR = 1.309, 95% CI: 1.062–1.614), lymph node metastasis (*P* = 0.001, HR = 1.620, 95% CI: 1.231–2.131), perineural invasion (*P* = 0.044, HR = 1.840, 95% CI: 1.017–3.332), CA19.9 (*P* = 0.029, HR = 1.423, 95% CI: 1.037–1.952) and sarcopenia (*P* = 0.0211, HR = 1.367, 95% CI: 1.049–1.782) were independent predictive factors (Table 4).

**Table 4:** Cox regression analysis was performed to verify independent predictive factors for overall survival

Characteristic	Univariate analysis		Multivariate analysis	
	*P* Value	HR(95.0% CI)	*P* Value	HR(95.0% CI)
Gender	0.363	0.890(0.692–1.144)		
Age	0.003	1.448(1.131–1.854)	0.000	1.622(1.258–2.091)
Tumour size	0.622	1.063(0.833–1.357)		
Location	0.003	0.745(0.611–0.907)	0.001	0.714(0.585–0.871)
Gross morphology	0.396	1.088(0.895–1.322)		
Tumour grading	0.006	0.345(0.163–0.732)		
Component	0.000	2.504(1.778–3.526)	0.001	1.799(1.263–2.561)
T	0.000	1.681(1.412–2.001)	0.012	1.309(1.062–1.614)
N	0.000	2.087(1.799–2.422)	0.001	1.620(1.231–2.131)
AJCC/UICC	0.000	2.269(1.856–2.774)		
Lymphovascular invasion	0.000	3.137(2.117–4.648)		
Perineural invasion	0.000	4.311(2.465–7.540)	0.044	1.840(1.017–3.332)
Chemotherapy	0.006	0.708(0.553–0.906)		
CEA	0.000	1.598(1.249–2.044)		
CA19.9	0.000	2.024(1.505–2.722)	0.029	1.423(1.037–1.952)
Sarcopenia	0.017	1.379(1.060–1.794)	0.0211	1.367(1.049–1.782)

Similarly, independent predictive factors for RFS were identified by the Cox regression analysis. Initially, univariate Cox regression was performed to identify variables significantly associated with RFS, revealing that age (*P* = 0.030, HR = 1.305, 95% CI: 1.026–1.659), tumour location (*P* = 0.008, HR = 0.774, 95% CI: 0.641–0.934), tumour grading (*P* = 0.009, HR = 0.390, 95% CI: 0.193–0.787), histological component (*P* < 0.001, HR = 2.195, 95% CI: 1.578–3.054), depth of invasion (*P* < 0.001, HR = 1.542, 95% CI: 1.311–1.813), lymph node metastasis (*P* < 0.001, HR = 2.136, 95% CI: 1.848–2.469), AJCC/UICC stage (*P* < 0.001, HR = 2.367, 95% CI: 1.940–2.889), lymphovascular invasion (*P* < 0.001, HR = 3.041, 95% CI: 2.079–4.449), perineural invasion (*P* < 0.001, HR = 3.878, 95% CI: 2.219–6.778), adjuvant chemotherapy (*P* < 0.001, HR = 0.623, 95% CI: 0.488–0.794), CEA (*P* < 0.001, HR = 1.617, 95% CI: 1.271–2.056), CA19.9 (*P* < 0.001, HR = 1.960, 95% CI: 1.460–2.631) and sarcopenia (*P* = 0.037, HR = 1.310, 95% CI: 1.017–1.689) were significantly associated with RFS (Table 5). Then, these variables that were significantly associated with RFS, as proven by the univariate Cox regression analysis, were included in the multivariate Cox regression analysis, revealing that age (*P* = 0.002, HR = 1.465, 95% CI: 1.145–1.873), tumour location (*P* = 0.002, HR = 0.743, 95% CI: 0.615–0.897), histological component (*P* = 0.015, HR = 1.536, 95% CI: 1.088–2.168), lymph node metastasis (*P* = 0.001, HR = 1.558, 95% CI: 1.194–2.034), CEA (*P* = 0.023, HR = 1.339, 95% CI: 1.041–1.722), CA19.9 (*P* = 0.026, HR = 1.421, 95% CI: 1.043–1.936) and sarcopenia (*P* = 0.045, HR = 1.299, 95% CI: 1.006–1.677) were independent predictive factors for RFS (Table 5).

**Table 5:** Cox regression analysis was performed to verify independent predictive factors for recurrence-free survival

Characteristic	Univariate analysis		Multivariate analysis	
	*P* Value	HR(95.0% CI)	*P* Value	HR(95.0% CI)
Gender	0.793	0.968(0.759–1.234)		
Age	0.030	1.305(1.026–1.659)	0.002	1.465(1.145–1.873)
Tumour size	0.629	1.061(0.835–1.346)		
Location	0.008	0.774(0.641–0.934)	0.002	0.743(0.615–0.897)
Gross morphology	0.674	1.042(0.860–1.263)		
Tumour grading	0.009	0.390(0.193–0.787)		
Component	0.000	2.195(1.578–3.054)	0.015	1.536(1.088–2.168)
T	0.000	1.542(1.311–1.813)		
N	0.000	2.136(1.848–2.469)	0.001	1.558(1.194–2.034)
AJCC/UICC	0.000	2.367(1.940–2.889)		
Lymphovascular invasion	0.000	3.041(2.079–4.449)		
Perineural invasion	0.000	3.878(2.219–6.778)		
Chemotherapy	0.000	0.623(0.488–0.794)		
CEA	0.000	1.617(1.271–2.056)	0.023	1.339(1.041–1.722)
CA19.9	0.000	1.960(1.460–2.631)	0.026	1.421(1.043–1.936)
Sarcopenia	0.037	1.310(1.017–1.689)	0.045	1.299(1.006–1.677)

## Discussion

Globally, CRC remains a huge health burden with remarkable recurrence risks. Factors resulting in sarcopenia and the possible reasons why sarcopenia could significantly affect short-term and long-term outcomes of patients with colorectal cancer have not been fully investigated. It has been hypothesized that sarcopenia identified by CT could be used as a reliable but modifiable factor for postoperative complications, deaths and cancer recurrence after curative surgery. By improving the nutritional status of patients before surgery, postoperative outcomes could be significantly improved [24, 25]. Left-sided colon cancer and rectal cancer are the most common types of CRC. It is known that left-sided colon cancer and rectal cancer are different from right-sided colon cancer in terms of postoperative complications and long-term survival. However, studies solely investigating the impacts of sarcopenia on short-term and long-term outcomes of patients with left-sided colon cancer or rectal cancer remain scarce. Thus, the present study was performed to investigate whether sarcopenia significantly affects the short-term and long-term outcomes of patients with left-sided colon cancer or rectal cancer.

First, it was demonstrated that the SG had a longer hospital stay after surgery, more intraoperative blood transfusions, and higher incidence of anastomotic fistula, SSI and hypoalbuminemia. Additionally both 30-day mortality and 90-day mortality for SG were also significantly higher than those for NSG. Second, Kaplan‒Meier analysis revealed that the patients in the SG had worse long-term survival and that sarcopenia was an independent predictive factor for both OS and RFS. To the best of our knowledge, the present study is one of the few studies systematically investigating the impacts of sarcopenia on the short-term and long-term outcomes of patients with left-sided colon cancer or rectal cancer after curative surgery.

The results of this study were consistent with those of some previous studies. In fact, sarcopenia has been reported as a risk factor for patients with various cancers, such as hepatocellular carcinoma [7], gastric cancer [9], bladder cancer [10], breast cancer [11], and pancreatic cancer [25]. A series of studies have investigated the impacts of preoperative sarcopenia on the short-term and long-term outcomes of patients with CRC. Deng CY et al. reported that progressive sarcopenia after the diagnosis of CRC had a significant negative prognostic association with overall and progression-free survival [26]. According to Trejo-Avila M et al., for patients with CRC, sarcopenia was a strong predictor of increased postoperative complications and worse survival outcomes [27]. In a study by Chai VW et al., sarcopenia was an objective, available predictive factor that is superior to the current biochemical and clinical measures of nutritional and functional status in predicting complications and cancer recurrence after curative resection for CRC [28]. Xie H et al. argued that preoperative CT-assessed sarcopenia could be employed as an effective predictor of complications and long-term prognosis for patients with CRC [29]. However, to date, there are no adequate studies investigating its significance among patients with left-sided colon cancer or rectal cancer. Therefore, our study could provide more knowledge into the significance of sarcopenia in CRC.

Despite the fact that sarcopenia has been proven to be a reliable predictor of short-term and long-term outcomes of patients with CRC, the reasons why sarcopenia leads to worse short-term and long-term outcomes have not been fully clarified. Sarcopenia has been proposed as a reflection of increased metabolic activity caused by more aggressive cancer biology, systemic inflammatory reaction and muscle depletion [30], which might in part explain why sarcopenia is a predictive factor for poorer prognosis. According to Hu WH et al., sarcopenia was significantly associated with higher IL-23 concentrations, and the combination of sarcopenia and IL-23 could more efficiently predict prognosis, suggesting that IL-23 and systemic inflammation were the possible mechanisms leading to worse survival [31]. However, the molecular mechanisms underlying sarcopenia are obviously complex, and it is almost impossible for us to raise both reproducible and sound explanations for all the recorded effects, the primary reason for which is that almost all components of the innate immune system are been involved in sarcopenia, including the catecholamine-cortisol system, chemokine signalling, interferons, complement cascades, immune-competent cells and other somatic cells [32]. These components of the innate system do not independently affect the occurrence of sarcopenia but communicate with and influence each other in a web-like manner [32]. Therefore, it is not easy for us to design persistently effective methods to reverse sarcopenia. However, some strategies have been proposed. First, daily exercise was recommended since it prevents the atrophy of muscle disuse. Additionally, it was reported that daily exercise would help reduce the concentrations of inflammation markers [32]. However, exercise is not always appropriate. Therefore, other interventions should be considered. Second, dietary or intravenous nutritional supplementation could improve nutritional status and then improve sarcopenia. Third, drugs targeting the inflammatory response are being investigated. However, their efficacy and persistence need to be improved. As a methylxanthine phosphodiesterase inhibitor drug initially used to treat asthma and COPD, theophylline has been investigated in alleviating systematic inflammation and sarcopenia [32]. However, the precise mechanisms through which theophylline alleviates sarcopenia remain to be determined. It was reported that after the application of theophylline, the production of pro-inflammatory IL-1, IL-6, IL-8 and TNF was significantly reduced [32–34]. Despite this finding, the application of theophylline among patients with sarcopenia caused by cancer should be prudent. Apart from theophylline, other potential drugs include infliximab, corticosteroids, thalidomide and related drugs, 4-aminoquinoline drugs (chloroquine, hydroxychloroquine and amodiaquine), nonsteroidal anti-inflammatory drugs, beta-adrenergic receptor blockers, statins, metformin, and hormonal treatment [32]. However, similar to theophylline, these drugs should be prudently used among patients with sarcopenia caused by cancer.

Kaplan‒Meier and Cox regression analyses demonstrated that preoperative sarcopenia was significantly associated with worse OS and RFS, which was consistent with previous studies. Dolan DR et al. reported that sarcopenia predicted worse 30-day mortality and 1-year survival [35]. According to Miyamoto Y et al., sarcopenia was a negative predictor for the survival of patients with stage I-III CRC undergoing curative surgery [13]. Furthermore, Takiguchi K et al. argued that preoperative sarcopenia was associated with prognosis and that prognosis would significantly improve after sarcopenia had been alleviated [36]. Additionally, they also reported that not only preoperative but also postoperative nutritional interventions were important for improving sarcopenia, which might thereby improve patient survival [36]. It was also reported by some other studies that short-term and long-term outcomes of patients with rectal cancer were negatively affected by sarcopenia [37–42].However, Sergei Bedrikovetski et al. reported that sarcopenia was not a predictor of poor neoadjuvant chemoradiotherapy response in locally advanced rectal cancer [43]. However, most studies reported the negative impacts of sarcopenia on short-term or long-term outcomes of patients with rectal cancer. Whereas studies assessing the impacts of sarcopenia on outcomes of patients with left-sided colon cancer have not been found. More studies were needed to further investigate the impacts of sarcopenia on left-sided colon cancer or rectal cancer. Takiguchi K et al. proposed several reasons why sarcopenia led to worse survival of patients with CRC. First, improved sarcopenia might increase muscle mass and activity, and better muscle activity was associated with better survival [36]. Second, improved sarcopenia was likely to result in a better course of other diseases, such as chronic heart failure, cirrhosis and chronic obstructive pulmonary disease [44–47]. The mortality risk due to these chronic diseases might be reduced after sarcopenia is improved. It seemed that the reduced mortality risk due to improved sarcopenia was attributable to the associations between sarcopenia and immune senescence and the fact that sarcopenia impaired cancer immunity [48–50]. Additionally, immunity against inflammation and the inflammatory tumour microenvironment are involved in the occurrence and progression of cancer [51–53]. For patients with extrahepatic cholangiocarcinoma, it was reported that sarcopenia negatively affected systemic and localized immune responses and led to poorer postoperative prognosis [54]. Third, sarcopenia leads to worse tolerance for various treatments, such as surgery, chemotherapy and radiotherapy, especially when cancer recurrence occurs. Moreover, treatment intensity significantly affects prognosis after cancer recurrence [55]. However, the treatment alternatives for individual patients should be decided by the physicians in charge after the physicians consider the patient’s general status, daily activity and organ functions. Additionally, sarcopenia also affects toxicity and reactivity after chemotherapy [56, 57]. Better nutritional status and improvements in sarcopenia when chemotherapy was applied were significantly associated with a better response and fewer severe side effects. Recently, immune checkpoint inhibitors (ICIs) have been widely used to treat cancers. Of these ICIs, programmed death-1 (PD-1) is the common target adopted in immune therapy. It was reported in non-small cell lung cancer that patients with sarcopenia had significantly poorer responses to programmed death-1 (PD-1) inhibitors [58, 59]. However, so far, there have been no studies investigating the impacts of sarcopenia on the responses of patients with CRC to PD-1 inhibitors. PD-1 inhibitors are commonly used among patients with high-frequency microsatellite instability. Thus, studies investigating the associations between sarcopenia and microsatellite status and the impacts of sarcopenia on the responses of patients with CRC to PD-1 inhibitors are warranted.

However, some shortcomings of this study should be discussed. First, this study was retrospective in nature, suggesting that selection bias was not absolutely avoidable. Therefore, prospective studies are needed to further verify the findings of this study. Second, a relatively small number of patients were included in this study, warranting studies with larger sample sizes. Third, since this study utilized data from a single country, the conclusions from this study should not be directly applied to patients from other countries since patients from different countries may have different backgrounds.

Despite these shortcomings of our study, it could still provide some guidance for future clinical practice. To the best of our knowledge, our study is one of the few studies assessing the impacts of sarcopenia on the short-term and long-term results of patients undergoing curative surgery. By improving sarcopenia and nutritional status, we could thereby remarkably improve their short- and long-term outcomes after curative surgery.

## Conclusion

In conclusion, preoperative sarcopenia identified on CT was an independent predictive factor for worse OS and RFS in patients with left-sided colon or rectal cancer after curative surgery. Preoperative CT scans are mandatory to assess whether sarcopenia exists since preoperative nutritional supplementation could improve short- and long-term outcomes.

## Data Availability

The datasets used and/or analyzed during the current study are available from the corresponding author on reasonable request.

## Abbreviations

PMI: Psoas muscle index
SG: Sarcopenia group
NSG: Nonsarcopenia group
BMI: Body mass index
OS: Overall survival
RFS: Recurrence-free survival
CI: Confidence interval
MDT: Multidisciplinary teamwork
CT: Computed tomography
HR: Hazard ratio
ASA: American Society of Anesthesiologists
CCI: Charlson Comorbidity Index
ICIs: Immune checkpoint inhibitors; surgical site infection (SSI)
PD-1: Programmed death-1
